# Clinical significance of coronavirus disease 2019 in hospitalized patients with myocardial injury

**DOI:** 10.1002/clc.23530

**Published:** 2021-01-27

**Authors:** Michael Briscoe, Robert Sykes, Thomas Krystofiak, Oliver Peck, Kenneth Mangion, Colin Berry

**Affiliations:** ^1^ Department of Cardiology Queen Elizabeth University Hospital, NHS Greater Glasgow and Clyde Health Board Glasgow UK; ^2^ Institute of Cardiovascular and Medical Sciences University of Glasgow Glasgow UK; ^3^ West of Scotland Heart and Lung Centre Golden Jubilee National Hospital Glasgow UK

**Keywords:** coronavirus, COVID‐19, myocardial Injury, outcomes, troponin, Type 2 myocardial infarction

## Abstract

**Background:**

The clinical significance of Coronavirus disease 2019 (COVID‐19) as an associate of myocardial injury is controversial.

**Hypothesis:**

Type 2 MI/Myocardial Injury are associated with worse outcomes if complicated by COVID‐19.

**Methods:**

This longitudinal cohort study involved consecutive patients admitted to a large urban hospital. Myocardial injury was determined using laboratory records as ≥1 hs‐TnI result >99th percentile (male: >34 ng/L; female: >16 ng/L). Endotypes were defined according to the Fourth Universal Definition of Myocardial Infarction (MI) and COVID‐19 determined using PCR. Outcomes of patients with myocardial injury with and without COVID‐19 were assessed.

**Results:**

Of 346 hospitalized patients with elevated hs‐TnI, 35 (10.1%) had laboratory‐confirmed COVID‐19 (median age [IQR]; 65 [59–74]; 64.8% male vs. COVID‐19 negative: 74 [63–83] years; 43.7% male). Cardiac endotypes by COVID‐19 status (yes vs. no) were: Type 1 MI (0 [0%] vs. 115 [100%]; *p* < .0005), Type 2 MI (13 [16.5%] vs. 66 [83.5%]; *p* = .045), and non‐ischemic myocardial injury (cardiac: 4 [5.8%] vs. 65 [94.2%]; *p* = .191, non‐cardiac:19 [22.9%] vs. 64 [77.%]; *p* < .0005). COVID‐19 patients had less comorbidity (median [IQR] Charlson Comorbidity Index: 3.0 [3.0] vs. 5.0 [4.0]; *p* = .001), similar hs‐TnI concentrations (median [IQR] initial: 46 [113] vs. 62 [138]; *p* = .199, peak: 122 [474] vs. 79 [220] ng/L; *p* = .564), longer admission (days) (median [IQR]: 14[19] vs. 6[12]; *p* = .001) and higher in‐hospital mortality (63.9% vs. 11.3%; OR = 13.2; 95%CI: 5.90, 29.7).

**Conclusions:**

Cardiac sequelae of COVID‐19 typically manifest as Non‐cardiac myocardial injury/Type 2MI in younger patients with less co‐morbidity. Paradoxically, the admission duration and in‐hospital mortality are increased.

## 1 INTRODUCTION

Coronavirus disease 2019 (COVID‐19) is a novel cause of myocardial injury.^1^ The importance of elevated cardiac troponin I (cTnI) in patients requiring intensive care has been extensively documented.^2^, ^3^ What is less well understood is the clinical significance of troponin elevation in COVID‐19 including in comparison with other causes of myocardial injury.

Cardiovascular involvement is observed in approximately 25% of patients with COVID‐19. Myocardial injury may be due to primary viral infection, secondary hypoxia, inflammatory state, hypotension, thromboembolism, or a combination of these problems.^4^ Mechanistic studies of the cardiac and multisystem effects of COVID‐19 infection are on‐going (ClinicalTrials.gov identifier NCT04403607).^5^ Non‐ischemic myocardial injury (cardiac or non‐cardiac) and Type 2 myocardial infarction (T2MI) generally portend an adverse prognosis, however, evidence‐based treatment options are less well established than is the case for Type 1 MI. Troponin‐I elevation is associated with fatal outcomes, however the etiology of this elevation is unclear..^6^

We prospectively investigated a population of consecutively admitted patients with myocardial injury in relation to COVID‐19 status. We hypothesized that in patients with myocardial injury, COVID‐19 would associate with Type 2 MI more so than with other endotypes, and this combination would be an adverse prognostic factor for in‐hospital outcomes.

## 2 METHODS

### 2.1 Study design

This was a prospective, longitudinal cohort study involving hospitalized patients admitted to the Queen Elizabeth University Hospital, Glasgow, UK; an urban academic medical center (catchment population 650 000) between March 1 and April 15, 2020. The study protocol and proforma were predefined and Caldicott guardian approval for the use of patient identifiable data was obtained before starting the project. Routinely collected (usual care) data were gathered by clinicians who were members of the usual care medical team and ethics approval or explicit patient consent was not required.

Consecutive patients who had a ≥1 hs‐TnI measurement (Abbott Architect TnI assay) >99th percentile sex‐specific upper reference limit (URL; male: >34 ng/L; female: >16 ng/L) were included in the cohort. The troponin‐I test was requested by the clinical team responsible for the patients' care based on clinical suspicion of myocardial injury by. The troponin test was not undertaken routinely on all admissions, nor on all patients with COVID‐19. A retrospective analysis of electronic patient records (NHS Greater Glasgow and Clyde: Clinical Portal, TrakCare systems) was performed. Source patient details, including demographic, routine laboratory and clinical data were evaluated in real‐time by a team of acute medical physicians (M.B., T.K., O.P., R.S.) and supervised by two senior cardiologists (K.M., C.B,). Index admissions for all patients ≥18‐years were considered; patients <18‐years or with incomplete or missing data were excluded.

Clinical endotypes of myocardial injury were defined according to the Fourth Universal Definition of Myocardial Infarction (MI) and sub‐categorized according to etiology by inciting event (cardiovascular and non‐cardiovascular). In case of diagnostic ambiguity, endotypes were assigned according to investigator consensus agreement following discussion with senior authors CB and KM.

### 2.2 Diagnosis of COVID‐19

A diagnosis of COVID‐19 was determined by either laboratory evidence of SARS‐CoV‐2 infection by PCR biospecimen testing and/or a radiological diagnosis (chest computed tomography, chest radiograph) consistent with COVID‐19 but biospecimen negative with a clinical presentation in keeping with SARS‐CoV‐2 infection. The laboratory tests used were either the Roche Cobas 6800 or Seegene SARS‐CoV‐2 tests.

### 2.3 Statistics

Patients were dichotomized by COVID‐19 status (positive or negative) and statistical analysis performed using IBM Statistics SPSS (version 24.0). Chi‐square (or Fisher's exact test) and odds ratios are calculated for categorical characteristic variables and Mann–Whitney U test for non‐parametric independent continuous variables to assess likelihood of association with COVID‐19 status. Sub‐group analyses were performed defined by clinical endotype of hs‐TnI elevation. Mortality and readmission significance were assessed by Log‐rank (Mantel Cox) test and 95% two‐tailed significance values provided. A *p*‐value of <.05 was considered significant.

## 3 RESULTS

**FIGURE 1:**
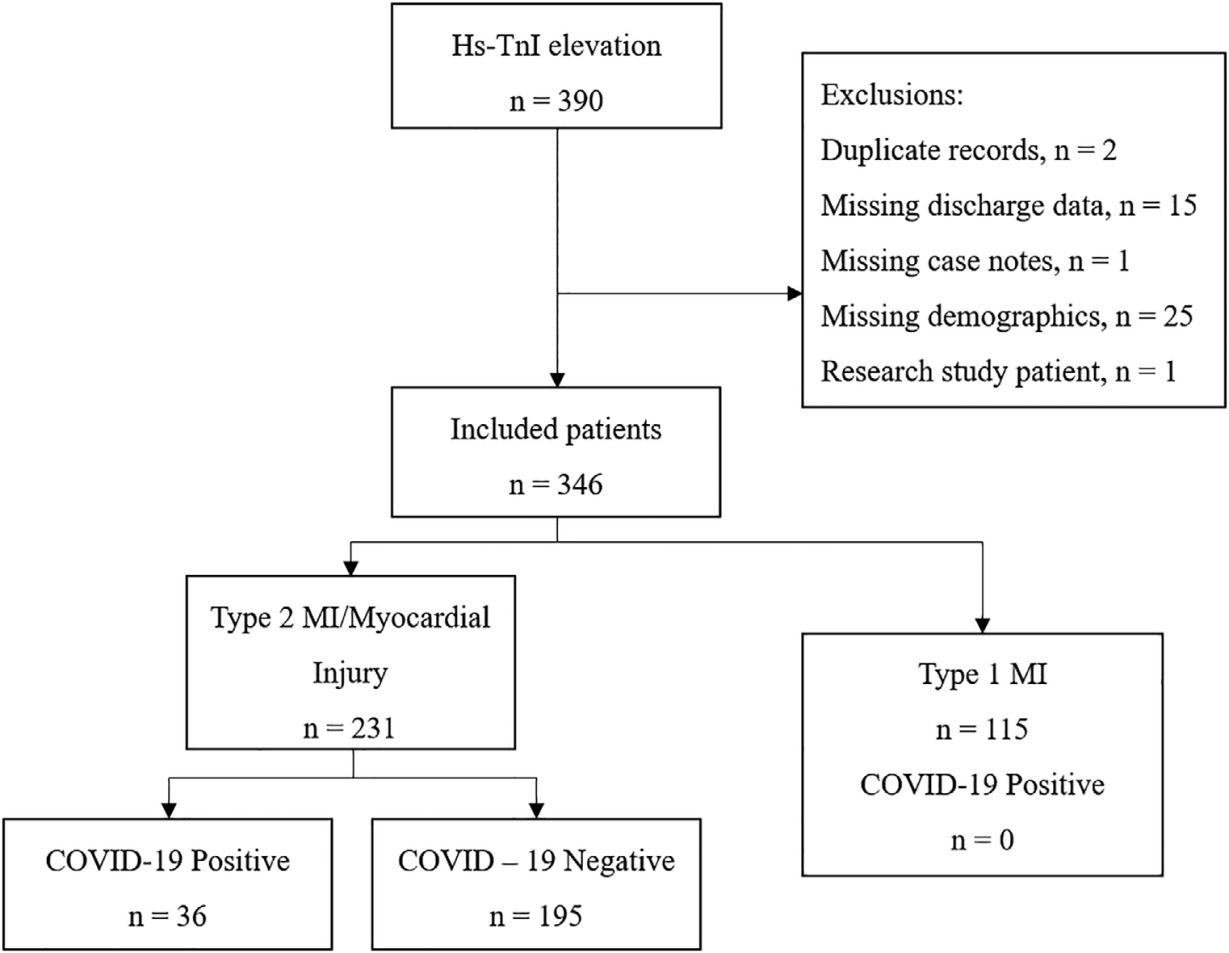
Flow diagram

Of 390 acutely hospitalized patients with elevated hs‐TnI between March 1–April 15, 2020, 346 were eligible for inclusion and 44 were excluded at screening (*Duplicates*: *2*; *Missing data*: *41*; *Research patient*: *1*). Type 1 myocardial infarction (T1MI) was diagnosed in 115 (33.2%) patients, Type 2 MI/non‐ischemic myocardial injury (non‐cardiac) was identified in 162 (46.8%) patients and myocardial injury of cardiac etiology in 69 (19.9%) patients (Figure 1). The distribution of endotypes within COVID positive and negative patients is presented in Tables S1 and S2.

Across all patient groups, 36 (10.4%) were diagnosed with COVID‐19. This included 35 (10.1%) laboratory‐confirmed COVID‐19 infection and one CT‐diagnosed patient admitted to COVID‐19 intensive care. The young PCR negative patient required invasive ventilation with peripherally‐predominant ground glass opacities throughout both lungs and negative microbiology and extended virology testing. Overall, this represented 38.7% of the inpatient laboratory‐confirmed COVID‐19 positive patients (*n* = 93) at our site during the study period. A combination of ECG findings and clinical assessment form the basis for diagnosis of Type 1 MI patients in our initial study population, of which no patients were concomitantly diagnosed with COVID‐19. The distributions of cardiac endotypes by COVID‐19 status (yes vs. no) were: Type 1 MI (0 [0%] vs. 115 [100%]; *p* < .0005), Type 2 MI (13 [16.5%] vs. 66 [83.5%]; *p* = .045), and non‐ischemic myocardial injury (cardiac: 4 [5.8%] vs. 65 [94.2%]; *p* = .191, non‐cardiac:19 [22.9%] vs. 64 [77.1%]; *p* < .0005). As no COVID‐19 positive patients were assigned a diagnosis of Type 1 MI, these were excluded from further analysis. In total, 232 patients with Type 2 MI (T2MI) or non‐ischemic myocardial injury (cardiac or non‐cardiac) were analyzed with dichotomization by COVID‐19 status.

### 3.1 Patient characteristics

The characteristics of patients with COVID‐19 infection and Type 2 MI or myocardial injury compared with those without COVID‐19 infection are described in Table 1. Among all patients, those with COVID‐19 were younger (median age [interquartile range]; 65 [59–74] vs. 74 [63–83] years) and 64.8% vs. 43.7% were male (*p* = .058). Patients with a diagnosis of COVID‐19 had comparable average hs‐TnI concentrations with COVID‐19 negative patients (median [interquartile range] initial hs‐TnI: 41 [106] vs. 61 [137] ng/L; *p* = .199, peak hs‐TnI: 121 [230] vs. 78 [204] ng/L; *p* = .564) (Table 1 and Table S4). Patients with COVID‐19 had a lower comorbidity burden (median [IQR] Charlson Comorbidity Index [CCI]: 3.0 [3.0] vs. 5.0 [4.0]; *p* = .001). Heart failure, chronic kidney disease (CKD), and chronic hematological disease were less common in patients with elevated cTnI and COVID‐19 infection; but obesity was significantly more prevalent when compared with COVID‐19 negative patients (44.4% vs. 14.9%, odds ratio [OR]: 4.58, 95% CI: 2.13, 9.86).

**TABLE 1:** Characteristics of hospitalized patients with myocardial injury defined by a high sensitivity troponin plasma concentration > URL described according to COVID‐19 status (Odds Ratio/Mean difference)

Variable	COVID +ve	COVID ‐ve	OR (95% CI)	*p* Value
(*N* = 232)	(*n* = 36)	(*n* = 195)		
Age ‐ median [IQR]	65.0 (5.0)	74.0 [20.0]	‐	.009
Female	13 (36.1%)	104 (53.3%)	0.50 (0.24, 1.03)	.058
Ethnicity^a^				.82
Arab	0 (0.0%)	1 (0.5%)		
South Asian	1 (2.8%)	2 (1.0%)		
West Asian	1 (2.8%)	5 (2.6%)		
White	34 (94.4%)	187 (95.9%)		
Died during admission (*n* = 46)	23 (63.9%)	23 ([11.3%)	13.9 (6.18, 31.3)	<.001
Overall mortality from admission to follow‐up (*n* = 60)	23 (63.9%)	37 (19.0%)	7.56 (3.50, 16.3)	<.001
Duration admission ‐ median [IQR]	14.0 (19.0)	6.0 (12.0)	‐	<.001
Survivors with unplanned readmission~ (*n* = 43)	3 (8.3%)	40 (23.1%)	0.35 (0.10, 1.21)	.104
Days until unplanned readmission (*n* = 43) ‐ median [range]	24 [22]	17.5 (64)	‐	.451
Initial troponin ‐ median [IQR]	41 [106]	61 [137]	‐	.199
Peak troponin ‐ median [IQR]	121 [230]	78 [204]	‐	.564
Past medical history				
Heart failure	7 (19.4%)	94 (48.2%)	0.26 (0.11, 0.62)	.001
Chronic pulmonary disease (not asthma)	10 (27.8%)	45 (23.1%)	1.28 (0.58, 2.86)	.543
Asthma	4 (11.1%)	25 (12.8%)	0.85 (0.28, 2.61)	.776
Chronic kidney disease	4 (11.1%)	74 (37.9%)	0.20 (0.07, 0.60)	.002
Liver disease	0 (0.0%)	4 (2.1%)	1.02 (1.00, 1.04)	1
Chronic neurological disorder	1 (2.8%)	10 (5.1%)	0.53 (0.07, 4.26)	1
Malignant Neoplasm	2 (5.6%)	28 (14.4%)	0.35 (0.08, 1.54)	.185
Chronic hematological disease	0 (0.0%)	10.3%	0.90 (0.85, 0.94)	.05
Obesity	16 (44.4%)	29 (14.9%)	4.58 (2.13, 9.86)	<.001
Diabetes mellitus	10 (27.8%)	47 (24.1%)	1.21 (0.54, 2.70)	.638
Connective tissue disorder	3 (8.3%)	10 (5.1%)	1.68 (0.44, 6.44)	.433
Dementia	2 (2.8%)	13 (6.7%)	0.40 (0.05, 3.16)	.702
History of smoking	22 (61.1%)	104 (53.3%)	1.38 (0.67, 2.84)	.389
Current	4 (11.1%)	39 (20.0%)	0.50 (0.17, 1.50)	.208
Previous	18 (50.0%)	65 (33.3%)	2.00 (0.98, 4.10)	.056
Never	14 (38.9%)	91 (46.7%)	0.73 (0.35, 1.50)	.389
Peripheral vascular disease	0 (0.0%)	2 (1.0%)	1.01 (1.00, 1.03)	1
Charlson comorbidity index ‐ median [IQR]	3.0 [3.0]	5.0 [4.0]	‐	.001
Charlson comorbidity index estimated 10‐year survival ‐ median [IQR]	77.0% (69.0%)	21.0% (77.0%)	‐	.001

^a^ None of the patients had Black, East Asian or Latin American ethnic backgrounds.

Respiratory failure requiring invasive mechanical ventilation (IMV) occurred in 20 (55.6%) patients with COVID‐19, whilst 14 (38.9%) and 17 (47.2%) required renal and/or circulatory support, respectively. Compared with patients who are COVID‐19 negative, there is no significant difference in initial or peak troponin‐I values independent of ventilation status, renal and/or circulatory support (Table 2). Troponin‐I values within the COVID‐19 positive were not significantly different for initial or peak hs‐TnI for patients requiring IMV compared with supplementary oxygen (O2) (Initial median [IQR] hs‐TnI: IMV = 33 [55] ng/L vs. O2 = 34 [50] ng/L, *p* = .559; Peak median [IQR]) hs‐TnI: IMV = 103.5 [133]) ng/L vs. O2 = 70.5 [114] ng/L, *p* = .183). COVID‐19 positive patients who required renal replacement therapy (RRT) had comparable initial hs‐TnI (RRT median [IQR] = 35.5 [74] ng/L vs. no RRT median [IQR] = 36 [43] ng/L, *p* = .986) and peak hs‐TnI (median [IQR] = 97 [243] ng/L vs. 69.5 [81] ng/L, *p* = .259).

**TABLE 2:** Median [IQR] high sensitivity troponin I values (ng/L) for hospitalized patients with increased troponin and COVID‐19 stratified by ventilation status and renal or circulatory support compared with COVID‐19 negative patients

	Initial hs‐TnI median [IQR]		*p* value	Peak hs‐TnI median [IQR]		*p* value
COVID negative	(*n* = 195)	62 [138]		(*n* = 195)	79 [220]	‐
COVID positive	(*n* = 33)	46 [113]	.199	(*n* = 36)	122 [474]	.564
Supplementary oxygen	(*n* = 30)	34 [50]	.213	(*n* = 30)	70.5 [114]	.635
Invasive mechanical ventilation	(*n* = 20)	33 [55]	.821	(*n* = 20)	103.5 [133]	.326
Inotropic support	(*n* = 17)	34 [53]	.683	(*n* = 17)	76 [186]	.610
Hemodialysis	(*n* = 14)	35.5 [74]	.986	(*n* = 14)	97 [243]	.259

*Note:* *p*‐value represents the comparison of COVID‐19 positive patients with Type 2 MI/Myocardial Injury and subgroups compared with COVID‐19 negative patients with Type 2 MI/Myocardial Injury.

### 3.2 12‐lead electrocardiogram (ECG)

Of all included patients, three did not have a 12‐lead ECG available for retrospective assessment on digital patient records of which one was COVID‐19 positive. This patient had a maximal troponin I of 43 ng/L (median 16 [IQR: 24.5]) during ITU admission on continuous 3‐lead ECG monitoring and categorized as non‐cardiac myocardial injury. The two patients in the COVID‐19 negative group without 12‐lead ECG were: (1) a dialyzed patient with superadded diagnosis of UTI and elevated troponin; and (2) an agitated patient with dementia and infective exacerbation of chronic airways disease who did not tolerate 12‐lead ECG assessment. In the remaining 343 patients from the initial cohort, ECGs were reviewed by emergency and internal physicians when obtained and uploaded to digital patient record for retrospective review by the study authors. Cardiology opinion was sought where clinically required by usual care teams but was not requested routinely for each patient. Senior authors (CB and KM) are experienced Cardiologists and any dubiety in diagnosis was explored with them, and consensus made if necessary.

The ECG features are described in Table S3, including patients with a diagnosis of Type 1 MI from the initial cohort.

Patients with Type 2 MI or myocardial injury, less frequently underwent further coronary assessment with either CT coronary angiography (*n* = 5) or invasive catheter angiography (*n* = 6). Cardiac MRI was performed in patients with a diagnosis of myocarditis in addition to clinical and ECG assessment.

### 3.3 Cardiac myocardial injury in COVID‐19

**FIGURE 2:**
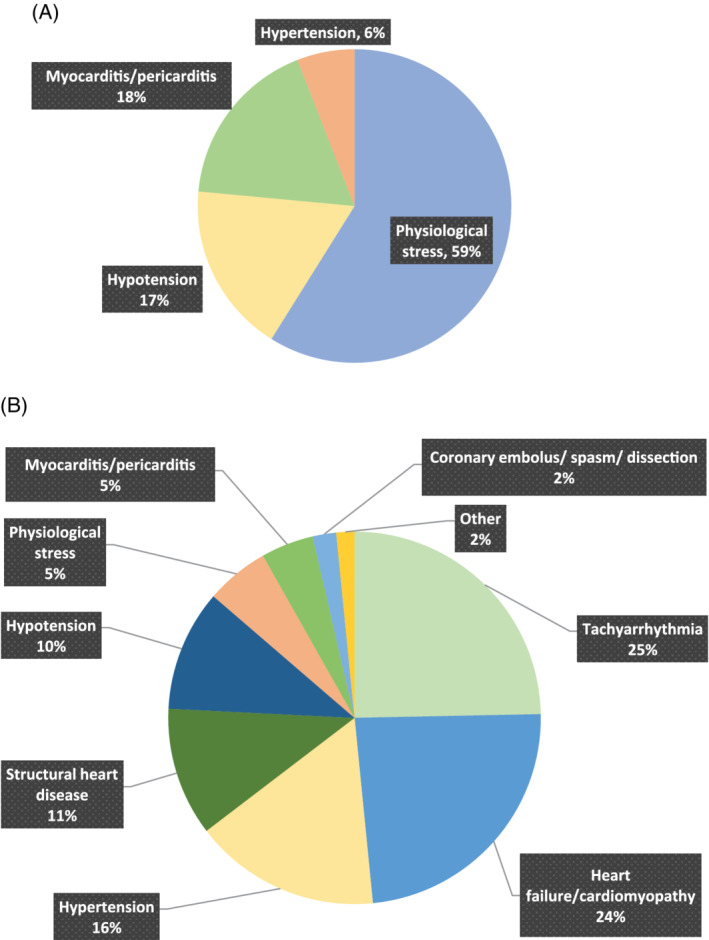
Proportion of cardiovascular causes of myocardial injury in COVID‐19 positive (A; *n* = 4) and negative (B; *n* = 65) patients

Physiological stress‐induced cardiac myocardial injury (i.e., hemodynamic shock, invasive ventilation, inotropic therapy) was the most prevalent cause for patients with COVID‐19 infection (27.8% vs. 3.5%, *p* < .001). Myocarditis/pericarditis was listed as causative in 8.3% COVID‐19 positive patients compared to 2.9% of all COVID‐19 negative patients (*p* = .116). Hypotension (8.3%) and hypertension (2.8%) were also implicated as causal associations for myocardial injury in the COVID‐19 positive patients, however these were not significant between groups (Figure 2 and Table S1). Cardiac causes of myocardial injury (Table S1) were more common in patients without COVID‐19 (positive: 11.1% vs. negative: 33.3%, *p* = .022), of which tachyarrhythmia and heart failure were the most likely mechanisms (15.7% and 15.1% respectively).

### 3.4 Non‐cardiac myocardial injury or Type 2 myocardial infarction in COVID‐19

**FIGURE 3:**
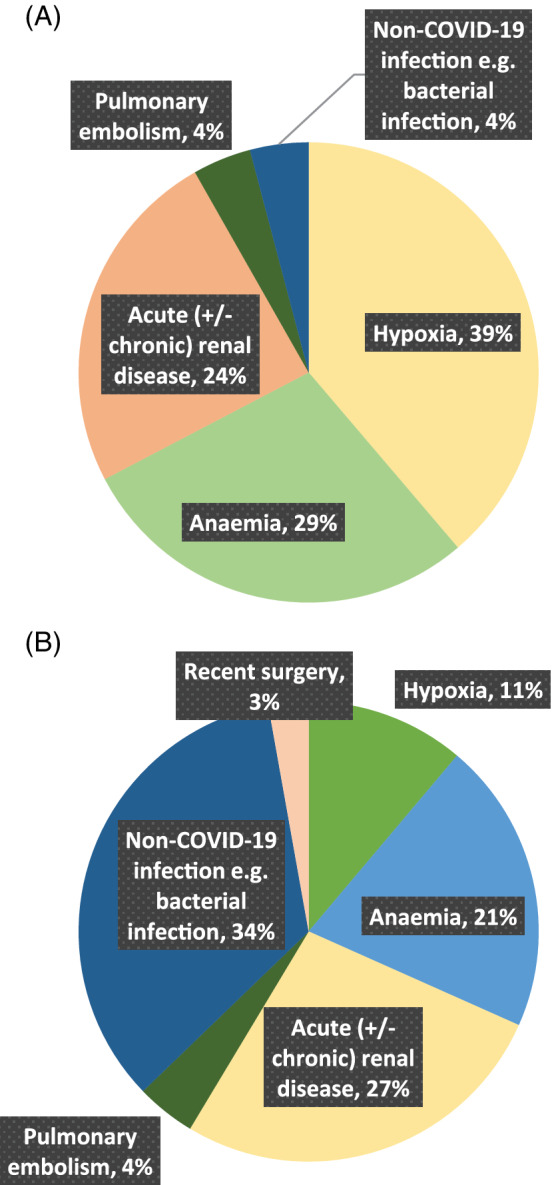
Proportion of Type 2 MI and non‐cardiac of myocardial injury in COVID‐19 positive (A; *n* = 32) and negative (B; *n* = 130) patients

Within this cohort, hypoxia was the most likely (OR: 7.96; 95% CI: 3.64, 17.4) contributing mechanism to non‐cardiac myocardial injury or Type 2 MI in patients with COVID‐19 (52.8%) compared with COVID‐19 negative patients (12.3%) (*p* < .001). Anemia was also significantly more likely in COVID‐19 positive patients (38.9% vs. 22.6%, OR: 2.18, 95% CI: 1.03, 4.62). Non‐COVID‐19 infection was the most common etiology in COVID‐19 negative patients (37.9%) and superadded infection in COVID‐19 positive patients was less common (5.6%) (OR: 0.1, 95% CI: 0.02, 0.41). Acute renal impairment (with or without underlying renal disease) was comparable between COVID status groups (positive: 33.3% vs. negative: 29.7%, *p* = .667) (Figure 3 and Table S2).

### 3.5 Outcomes of patients with elevated cTnI in COVID‐19

Vital status was determined in all patients by assessment of electronic patient records. The duration of follow‐up was a median of 71 days (interquartile range: 29.3, minimum: 0, maximum: 151 days) from date of admission. Overall, 73 (21%) patients died within this period. Patients who tested positive for COVID‐19 with elevated cTnI had a longer hospital stay (median [IQR]: 14 [19] vs. 6 [12]; *p* < .001) and higher inpatient mortality (63.9% vs. 11.3%; OR = 13.2; 95% CI: 5.90, 29.7) compared with negative patients. Index CCI 10‐year survival estimates were greater in patients with COVID‐19 compared with COVID‐19 negative patients (77.0% vs. 21.0% alive at 10 years, *p* < .001). Unplanned readmissions occurred in 43 patients in all groups however there was no significant difference in frequency (positive: 8.3% vs. negative: 20.5%, *p* = .104) or duration until readmission (median = positive: 24.0 [IQR: 22.0] vs. negative: 17.5 [IQR: 64.0]; *p* = .292) between groups.

## 4 DISCUSSION

We have provided novel information on the nature and clinical significance of COVID‐19 in a hospitalized population with myocardial injury.

Our study involved prospective clinical evaluation of consecutive patients in real‐time during the study period. Cases of myocardial injury were identified based on a high sensitivity troponin result >URL. This approach was intended to minimize missing data and selection bias. We found that the cardiac sequelae of COVID‐19 typically present as Type 2 MI or non‐cardiac myocardial injury; no patients presented with COVID‐19 infection and Type 1 MI during the study period. Compared to patients with myocardial injury without COVID‐19, patients with COVID‐19 and myocardial injury were younger and had fewer concomitant health problems (including a lower Charlson Comorbidity Index). By contrast, they had significantly greater in‐hospital mortality and a longer inpatient stay despite comparable hs‐TnI at baseline and peak values.

As has been widely reported, obesity is significantly associated with COVID‐19 and T2MI/non‐ischemic myocardial injury as opposed to non‐COVID‐19 etiologies of hs‐TnI elevation. Troponin elevation in a hospitalized population with COVID‐19 may provide information on disease severity and the poorer outcomes observed in this study are in keeping with existing literature (6). Non‐COVID‐19 etiologies included bacterial infection, tachyarrhythmia and heart failure. Hypoxia was the most likely contributing mechanism in patients with COVID‐19 in keeping with the common respiratory manifestations of this disease. Anemia was also more common in COVID‐19 patients with myocardial injury/Type 2 MI. There was no significant difference in incidence of pulmonary embolism, impaired renal status or presence of diabetes in this cohort of patients with myocardial injury/Type 2 MI regardless of COVID‐19 status however, the small number of troponin positive patients with these factors limits power in this study to detect a significant difference.

### 4.1 Implications for etiology of myocardial injury/Type 2 MI in COVID‐19 positive patients

We found that the hs‐TnI concentrations were broadly similar in the COVID‐19 and non‐COVID‐19 subgroups, although in‐hospital mortality was greater in COVID‐19 positive patients. In the context of COVID‐19, Type 2 MI/Myocardial injury was observed in younger, less co‐morbid patients. Hypoxia, anemia and renal dysfunction are also clearly relevant complications. This is in keeping with emerging autopsy reports of multi‐organ failure due to septic shock with respiratory infection being most common cause of death.^7^

There were no instances of tachyarrhythmia documented as the cause of hs‐TnI elevation in the 36 patients with COVID‐19 however three positive patients were noted to have existing atrial fibrillation with a maximum 12‐lead ECG rate of 131 bpm. Since continuous ECG monitoring data were not available, this finding may represent an under‐estimate. In prior studies, arrhythmia may be present in up to 44% of patients with severe illness. Case reports from other pandemic regions have included arrhythmias in the peri‐mortem stage of illness or secondary to hypoxemia mediated conduction deficits.^8^, ^9^ Further information on 12‐lead ECG observations in positive and negative patients is provided in Table S3.

Pericarditis/Myocarditis was recorded as the mechanism of myocardial injury in three patients with COVID‐19. Diagnosis was made on clinical suspicion with ECG changes and cardiac MRI as objective confirmation of diagnosis. Myocarditis in COVID‐19 has been reported previously – although uncommonly – and the results of prospective imaging studies will potentially illuminate the prevalence in surviving patients following COVID‐19 infection.^10^

Obesity was the most common co‐morbidity within our cohort, observed among 44.4% of patients with COVID‐19 and Type 2 MI/myocardial injury. Obesity is associated with adverse outcomes in COVID‐19 infection.^11^, ^12^, ^13^ A combination of increased cardiac demand and abnormalities of diastolic function in combination with hypoventilation in respiratory illness and high prevalence of metabolic disease are likely contributors..^14^, ^15^

Type 2 MI/myocardial injury is associated with increased illness severity as evidenced by significantly higher mortality and duration of hospital stay.^16^ Though exact mechanisms of myocardial injury and modifiable outcome in patients with COVID‐19 is yet to be demonstrated, protocolized high‐sensitivity troponin testing and its utilization in stratification could be a useful tool for early recognition of adverse outcome and early decision making in this group.^17^

### 4.2 Study limitations

Limitations of this study included a the moderate number of patients with COVID‐19 and concomitant hs‐TnI elevation within our population despite sampling during the first peak phase of disease activity in our hospital. The limited number of COVID‐19 patients with troponin I may have an impact on the statistical power to detect differences in hs‐TnI values, that is, in patients requiring renal replacement therapy with COVID‐19 had an approximately 30% greater value (ng/L) but this was not statistically significant. Troponin I is not routinely sampled in patients who present with COVID‐19 symptoms hence there may be an under‐representation of troponin elevation. Physiological stress and myocarditis/pericarditis were documented as the most prevalent causes of non‐ischemic cardiovascular myocardial injury in patients positive for COVID‐19. Advanced cardiovascular imaging (cardiac magnetic resonance imaging [CMRI], CT coronary angiography [CTCA]) was not routinely feasible on logistical grounds during the pandemic which may have led to a degree of under‐reporting of Type 1 MI.

### 4.3 Conclusions

The occurrence of myocardial injury and COVID‐19 is associated with a high in‐hospital mortality. Type 2 MI or myocardial injury in COVID‐19 infection has poorer in‐hospital outcomes compared with other causes of Type 2 MI or myocardial injury.

## CONFLICT OF INTEREST

Colin Berry is employed by the University of Glasgow, which holds consultancy and research agreements with companies that have interests in the diagnosis and treatment of angina. The companies include Abbott Vascular, AstraZeneca, Boehringer Ingelheim, Coroventis, GSK, HeartFlow, Medyria, Neovasc, Novartis and Siemens Healthcare.

The authors declare no potential conflicts of interest.

## Supporting information


**Appendix** **S1.** Supporting Information.

## Data Availability

The data that support the findings of this study are available on request from the corresponding author. The data are not publicly available due to privacy or ethical restrictions.
