# Supplementary material for: Clinical significance of coronavirus disease 2019 in hospitalized patients with myocardial injury
Source: Clin Cardiol. 2021 Jan 27;44(3):332–9. doi: 10.1002/clc.23530 (PMC7943910; doi:10.1002/clc.23530)
Supplement: Supplementary file 1 — Appendix S1. Supporting Information. [file CLC-44-332-s001.docx]

Supplement

Clinical Significance of Coronavirus Disease 2019 (COVID-19) in Hospitalized Patients with Myocardial Injury

## Supplementary Table A – Cardiovascular causes of Myocardial Injury.

|  | **COVID +ve**  **(n = 4)** | **COVID -ve**  **(n = 65)** | **P value** |
| --- | --- | --- | --- |
| **Cardiac Cause** | **11.1%** | **33.3%** | **0.022** |
| Stress | 27.8% | 3.5% | <0.001 |
| Hypotension | 8.3% | 6.7% | 0.726 |
| Myocarditis/pericarditis | 8.3% | 2.9% | 0.116 |
| Hypertension | 2.8% | 10.3% | 0.228 |
| Tachyarrhythmia | 0.0% | 15.7% | 0.005 |
| Coronary embolus/ spasm/ dissection | 0.0% | 1.3% | 1.0 |
| Heart failure/cardiomyopathy | 0.0% | 15.1% | 0.008 |
| Structural heart disease | 0.0% | 7.1% | 0.147 |
| Other | 0.0% | 1.0% | 1.0 |

## Supplementary Table B – Type 2 MI and Non-Cardiac Causes of Myocardial Injury.

|  | **COVID +ve**  **(n = 32)** | **COVID -ve**  **(n = 130)** | **P value** |
| --- | --- | --- | --- |
| **Type 2 MI/Non-cardiac myocardial injury (n = 161)** | **88.9%** | **66.6%** | **0.022** |
| Hypoxia | 52.8% | 12.3% | <0.001 |
| Anaemia | 38.9% | 22.6% | 0.038 |
| Acute (+/- chronic) renal disease | 33.3% | 29.7% | 0.667 |
| Pulmonary embolism | 5.6% | 4.6% | 0.683 |
| Non-COVID-19 infection e.g. bacterial infection | 5.6% | 37.9% | <0.001 |
| Recent surgery | 0.0% | 3.1% | 0.594 |

**Supplementary Table C – 12-lead ECG data from included patients.**

|  | COVID Positive  (n = 36) | COVID negative*  (n = 195) | Type 1 MI  (n = 115) |
| --- | --- | --- | --- |
| Atrial Fibrillation | 3 | 38 | 7 |
| Sinus | 32 | 143 | 106 |
| Ventricular tachycardia | 0 | 2 | 1 |
| Complete Heart Block | 0 | 5 | 0 |
| Paced rhythm | 0 | 5 | 1 |
| Missing ECG | 1 | 2 | 0 |
|  |  |  |  |
| ST Elevation | 0 | 6 | 26 |
| ST Depression | 3 | 35 | 20 |
| T-wave inversion | 4 | 63 | 36 |
| Isolated ST Elevation aVR | 0 | 8 | 2 |
| Left bundle branch block | 2 | 19 | 7 |
| Right bundle branch block | 6 | 18 | 6 |
| QRS (IQR) - ms^-1^ | 92 (18) | 92 (28) | 92 (16.5) |
| Rate (IQR) - bpm | 91 (20.5) | 86 (38) | 78 (22) |
| Max rate - bpm | 131 | 196 | 204 |
| Min rate - bpm | 44 | 36 | 45 |
|  |  |  |  |
| New ST changes, arrhythmia or conduction defect | 2 | 56 | 73 |
| Serial ECGs available | 24 | 148 | 55 |

*COVID-19 negative group not including patients with Type 1 MI.

**Supplementary Table D – Average troponin I values across all study participants.**

| Troponin I (ng/L) | COVID Positive | COVID negative* | Type 1 MI |
| --- | --- | --- | --- |
| Average Initial [IQR] | 41 [106] | 61 [137] | 306 [3,001] |
| Average Peak [IQR] | 121 [230] | 78 [204] | 946 [9,284] |
|  |  |  |  |
| Minimum* | <4 | <4 | <4 |
| Quartile 1 | 18 | 33 | 72 |
| Quartile 2 | 45 | 69 | 306 |
| Quartile 3 | 154 | 213 | 3,073 |
| Maximum* | 7,171 | 13,856 | >50,000 |

*****Minimum detectable value of <4 ng/L and maximum value of >50,000 ng/L
